# Prognostic value of laboratory markers and clinical scores for mortality in intensive care unit patients with sepsis

**DOI:** 10.1371/journal.pone.0337396

**Published:** 2025-12-04

**Authors:** So-yun Kim, Dukki Kim, Hyekyeong Ju, Song I. Lee

**Affiliations:** Division of Pulmonary and Critical Care Medicine, Department of Internal Medicine, Chungnam National School of Medicine, Chungnam National University Hospital, Daejeon, Republic of Korea; Macquarie University / George Institute for Global Health, AUSTRALIA

## Abstract

**Introduction:**

Sepsis is a life-threatening condition, especially for patients in the intensive care unit (ICU), where early identification of the prognosis is critical. This study aimed to evaluate the prognostic value of inflammatory markers, clinical scores, and specific laboratory findings for predicting ICU and in-hospital mortality in sepsis patients.

**Methods:**

A retrospective cohort study was conducted on adult patients with sepsis who were admitted to the ICU of a university hospital between September 2019 and December 2022. To minimize selection bias, all eligible patients during the study period were consecutively included. Data were extracted from electronic medical records and included demographics, clinical characteristics, inflammatory markers, and clinical scores such as the Charlson Comorbidity Index (CCI), Clinical Frailty Scale (CFS), Eastern Cooperative Oncology Group (ECOG) performance status, Simplified Acute Physiology Score 3 (SAPS 3), and Sequential Organ Failure Assessment (SOFA). The primary outcomes were ICU and in-hospital mortality. Univariate and multivariate Cox regression analyses were performed to identify predictors of mortality.

**Results:**

A total of 213 ICU patients with sepsis were included in the study. The patients were 62.0% male with a mean age of 73.1 ± 12.6 years. The ICU and in-hospital mortality rates were 29.6% and 36.6%, respectively. Non-survivors had higher clinical severity scores and poorer nutritional and perfusion profiles than survivors. Multivariate analysis revealed that elevated lactate levels (a marker of tissue hypoperfusion) and higher SAPS 3 scores were independently associated with ICU mortality. For in-hospital mortality, lower albumin levels and higher SAPS 3 scores were significant predictors.

**Conclusions:**

High lactate and SAPS 3 scores were independent predictors of mortality, while higher albumin levels showed a potential protective effect. Early identification of these factors may aid in the management of sepsis.

## Introduction

Sepsis is a serious medical emergency caused by the body’s extreme response to infection, often resulting in widespread inflammation, tissue damage, and organ failure [1]. Early identification and appropriate management of patients with sepsis, particularly in intensive care unit (ICU) patients, are crucial for improving clinical outcomes [2]. The heterogeneity and rapid progression of sepsis necessitate reliable predictors of patient prognosis to guide timely and effective treatment.

Laboratory biomarkers reflecting host inflammatory and immune responses play a key role in the assessment of sepsis [3]. Common markers such as white blood cell (WBC) count, neutrophil-lymphocyte ratio (NLR), platelet count, platelet-to-lymphocyte ratio (PLR) [4] and C-reactive protein (CRP) [5] are widely used to assess systemic inflammation. More specific markers, including procalcitonin and presepsin [6], can help differentiate sepsis from other inflammatory conditions. In addition, lactate levels [7], which indicate tissue hypoperfusion, are critical in assessing the severity of septic shock and predicting outcome.

In addition to individual inflammatory markers, comprehensive scoring systems have been developed to assess the overall health status and prognosis of patients with sepsis. The Charlson Comorbidity Index (CCI) assesses the burden of chronic diseases and provides long-term prognostic insight [8]. The Clinical Frailty Scale (CFS) assesses patient frailty and provides insight into their ability to recover from severe illness [9]. The Eastern Cooperative Oncology Group (ECOG) Performance Status measures patients’ overall well-being and activity level [10]. The Simplified Acute Physiology Score 3 (SAPS 3) [11] and Sequential Organ Failure Assessment (SOFA) [12] scores are widely used in the ICU to predict mortality and monitor organ dysfunction in patients with sepsis.

While numerous studies have examined the prognostic value of inflammatory markers or clinical scoring systems individually, limited research has examined their combined utility in predicting outcomes in ICU patients with sepsis. Given the complexity of sepsis and the diversity of patient presentations, integrating biomarker data with clinical scores may improve risk stratification and support more tailored therapeutic decision-making. Therefore, the aim of this study was to evaluate the prognostic value of selected inflammatory markers, laboratory findings, and clinical scoring systems in predicting ICU and hospital mortality in patients with sepsis. By combining these parameters, we aimed to provide a more comprehensive prognostic model that could facilitate early identification of high-risk patients and improve clinical management in the ICU.

## Materials and methods

### Patients and data collection

**Fig 1:**
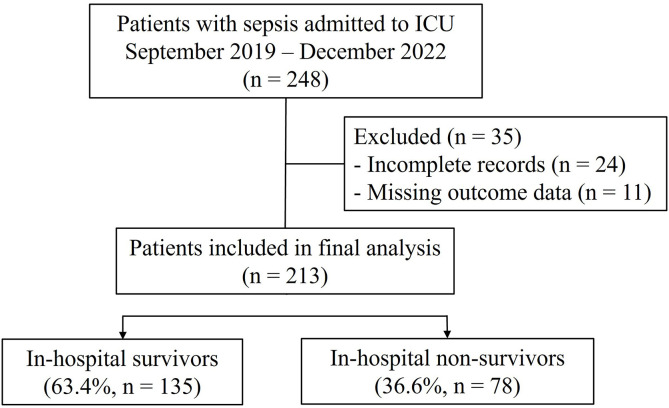
Flowchart of patient inclusion and exclusion.

This retrospective cohort study involved adult patients (aged 19 years and older) diagnosed with sepsis and was conducted at a 1,000-bed, university-affiliated tertiary care hospital is located in Daejeon, South Korea. The hospital has multiple intensive care units (ICU) managed by board-certified intensivists, including a 16-bed medical ICU, a 14-bed surgical ICU, a 20-bed emergency ICU, a 10-bed cardiac ICU, and a 20-bed neurological ICU. Patients with sepsis admitted to any of these ICUs between September 2019 and December 2022 were consecutively enrolled and observed until discharge or death to minimize selection bias. Data for this study were accessed on March 15, 2023 (initial access) and March 20, 2024 (final extraction). Patients with incomplete or missing medical records were excluded at the time of enrollment. The screening and enrollment process is summarized in the flow chart (Fig 1).

We reviewed the hospital’s electronic medical records and collected information on the patients’ baseline characteristics, age, body mass index (BMI), laboratory findings, sepsis characteristics, medication use, interventions, CCI, CFS, ECOG score, SAPS 3 score, SOFA score, ICU and hospital length of stay, and prognosis.

This study was approved by the Institutional Review Board (IRB) of Chungnam National University Hospital (IRB number: 2021-07-041-008). As the study was retrospective in design, the IRB waived the requirement for informed consent from participants.

### Definitions and outcomes

Sepsis was defined according to the Sepsis-3 criteria [1] as life-threatening organ dysfunction due to dysregulated host response to infection, with a SOFA score elevation of 2 or more points. The CCI [13] was used to assess the chronic disease burden. The CFS [14] assesses a patient’s frailty status on a scale of 1–9. The ECOG performance status [15] measured the patient’s ability to perform activities of daily living on a scale of 0 (fully active) to 5 (deceased). The SAPS 3 score [16], which predicts in-hospital mortality based on several physiological and clinical parameters, was used to assess the severity of illness in ICU patients. Additionally, the SOFA score [17] was calculated using the worst values on the day of ICU admission.

Inflammatory markers measured on the first day of ICU admission included WBC count, NLR, PLR, CRP, procalcitonin, presepsin, and lactate. The NLR was calculated by dividing the number of neutrophils by the number of lymphocytes, whereas the platelet-lymphocyte ratio was calculated by dividing the number of platelets by the number of lymphocytes.

The primary outcomes were ICU and in-hospital mortality. ICU mortality was defined as death from any cause during the initial ICU admission. In-hospital mortality was defined as death from any cause during the same hospitalization, regardless of ICU discharge status.

### Sample size calculation

For this survival analysis, sample size adequacy was assessed based on the number of outcome events rather than the total sample size. According to the established guidelines for Cox proportional hazards modeling, there should be a minimum of ten outcome events per predictor variable to reduce the risk of overfitting and ensure model stability. In our cohort, there were 78 in-hospital deaths among the 213 enrolled patients, which allowed us to include 7–8 covariates in the multivariable model. Although initial recruitment expectations were based on conventional sample size calculations for binary outcomes, the final number of events was sufficient for robust survival analysis using Cox regression.

### Statistical analysis

We analyzed patient characteristics and clinical outcomes according to in-hospital survival status. Categorical variables are presented as counts and percentages, while continuous variables are expressed as means with standard deviations (SD) or medians with interquartile ranges (IQR), depending on the data distribution. We performed group comparisons between survivors and non-survivors using chi-square tests for categorical variables and Welch’s t-tests for continuous variables.

To identify predictors of ICU mortality, we performed univariate and multivariate Cox proportional hazards regression analyses. Time-to-event was defined as the number of days from ICU admission to ICU death. For survivors, follow-up was censored at ICU discharge. For the analysis of in-hospital mortality, time-to-event was defined as the number of days from ICU admission to hospital death, with censoring at hospital discharge. The median follow-up duration was 7.0 days (interquartile range [IQR]: 3.0–15.0) for ICU outcomes and 16.0 days (IQR: 8.0–33.0) for hospital outcomes.

Variables for the multivariate analysis were selected based on clinical relevance and statistical significance in the univariate analysis (p < 0.10). We assessed the proportional hazards assumption using scaled Schoenfeld residual tests (cox.zph() function in R). A marginal violation was noted for lactate in the ICU mortality model (p = 0.028), with a borderline significant global test (p = 0.033). No violations were observed in the in-hospital mortality model (all p > 0.05). Visual inspection of residual plots confirmed acceptable model assumptions.

We performed receiver operating characteristic (ROC) curve analysis to determine the optimal cutoff values for the selected continuous variables using Youden’s index. Kaplan-Meier survival curves were constructed using these cutoffs, and survival distributions were compared using log-rank tests. Hazard ratios (HR) and 95% confidence intervals (CI) were calculated from Cox models.

We conducted statistical analyses using SPSS version 25.0 (IBM Corp.) for regression analyses, GraphPad Prism version 9.0 (GraphPad Software) for survival curves, and R version 4.3.0 for assumption testing.

## Results

### Basic characteristics of patients

A total of 213 patients with sepsis who were admitted to the ICU were included in this study. Of these, 63.4% (n = 135) were in-hospital survivors, and 36.6% (n = 78) were in-hospital non-survivors.

Table 1 presents the patients’ basic characteristics. The mean age of all patients was 73.1 ± 12.6 years. Non-survivors were older than survivors (75.9 ± 11.2 vs. 71.5 ± 13.1 years, p = 0.011). In addition, BMI was lower in non-survivors compared to survivors (20.9 ± 3.9 vs. 23.2 ± 5.9, p = 0.003). Regarding underlying diseases, heart failure was slightly more prevalent in non-survivors than in survivors (9.0% vs. 3.0%, p = 0.056), and connective tissue disease was more prevalent in non-survivors than in survivors (9.0% vs. 3.0%, p = 0.056). There were no significant differences in the other underlying diseases between the two groups. The most common site of sepsis was the pulmonary tract (50.2%), followed by the urinary tract, abdomen, skin/soft tissue, and other sites. Pulmonary infections were more common in non-survivors than in survivors (73.1% vs. 37.0%, P < 0.001), whereas urinary tract infections were less common in non-survivors than in survivors (12.8% vs. 40.0%, P < 0.001).

**Table 1:** Baseline Characteristics of Enrolled patients.

Characteristics	All patients	Survivor	Non-survivor	P-value
Patients (n)	213	135	78	
**Age, yr**	73.1 ± 12.6	71.5 ± 13.1	75.9 ± 11.2	0.011
Male	132 (62.0)	79 (58.5)	53 (67.9)	0.172
Body mass index, kg/m^2^	22.4 ± 5.4	23.2 ± 5.9	20.9 ± 3.9	0.003
Community-acquired infection	177 (83.1)	117 (86.7)	60 (76.9)	0.068
Hospital-acquired infection	36 (16.9)	18 (13.3)	18 (23.1)	0.068
Underlying disease				
Diabetes Mellitus	79 (37.1)	52 (38.5)	27 (34.6)	0.570
Chronic kidney disease	28 (13.1)	14 (10.4)	14 (17.9)	0.115
Liver disease	15 (7.0)	11 (8.1)	4 (5.1)	0.407
Heart failure	11 (5.2)	4 (3.0)	7 (9.0)	0.056
Chronic obstructive lung disease	7 (3.3)	5 (3.7)	2 (2.6)	0.653
Cerebrovascular disease	65 (30.5)	41 (30.4)	24 (30.8)	0.951
Dementia	51 (23.9)	30 (22.2)	21 (26.9)	0.439
Connective tissue disease	11 (5.2)	4 (3.0)	7 (9.0)	0.056
Solid tumor	28 (13.1)	16 (11.9)	12 (15.4)	0.462
Hematologic malignancy	7 (3.3)	6 (4.4)	1 (1.3)	0.212
Suspected infection site of sepsis				
**Pulmonary**	107 (50.2)	50 (37.0)	57 (73.1)	<0.001
Abdomen	38 (17.8)	28 (20.7)	10 (12.8)	0.146
**Urinary tract**	64 (30.0)	54 (40.0)	10 (12.8)	<0.001
Skin/soft tissue	20 (9.4)	13 (9.6)	7 (9.0)	0.874
Other site	12 (5.6)	8 (5.9)	4 (5.1)	0.808

Data are presented as mean ± standard deviation or number (%) unless otherwise indicated.

Other sites: catheter-related, Systemic infection without clear primary site of infection, Neurologic

### Laboratory findings and patient scores

Table 2 shows the patients’ laboratory findings and scores. Non-survivors had higher scores than survivors on several indices: CCI (5.8 ± 2.5 vs. 5.1 ± 2.6, p = 0.039), CFS (6.1 ± 1.5 vs. 5.4 ± 1.6, p = 0. 002), ECOG performance status (2.8 ± 0.9 vs. 2.4 ± 1.0, p = 0.003), SAPS 3 (79.2 ± 13.1 vs. 68.3 ± 12.1, p < 0.001) and SOFA (8.2 ± 3.2 vs. 7.1 ± 3.0, p = 0.009). In terms of laboratory findings, non-survivors had lower albumin levels (2.6 ± 0.6 vs. 2.9 ± 0.6, p < 0.001) and higher lactate levels (4.42 ± 3.53 vs. 3.49 ± 2.58, p = 0.044) compared to survivors. Other laboratory values did not show any statistically significant differences.

**Table 2:** Patients’ laboratory findings and scores according to survivor.

Characteristics	All patients	Survivor	Non-survivor	P-value
Patients (n)	213	135	78	
Scores				
**Charlson Comorbidity index**	5.3 ± 2.6	5.1 ± 2.6	5.8 ± 2.5	0.039
**Clinical frailty scale**	5.6 ± 1.6	5.4 ± 1.6	6.1 ± 1.5	0.002
**ECOG performance status**	2.6 ± 1.0	2.4 ± 1.0	2.8 ± 0.9	0.003
**SAPS3 score**	72.3 ± 13.5	68.3 ± 12.1	79.2 ± 13.1	<0.001
**SOFA score**	7.5 ± 3.1	7.1 ± 3.0	8.2 ± 3.2	0.009
Initial laboratory data				
White blood cell, × 10^3^/uL	14.15 ± 9.95	14.62 ± 9.56	13.35 ± 10.60	0.370
Neutrophil lymphocyte ratio	22.6 ± 22.4	23.9 ± 22.8	20.4 ± 21.7	0.274
Hemoglobin, g/dL	11.1 ± 2.4	11.1 ± 2.3	11.0 ± 2.7	0.728
Platelet, × 10^3^/uL	77.0 ± 101.9	76.6 ± 101.0	77.8 ± 104.0	0.934
Platelet lymphocyte ratio	164.5 ± 393.0	164.3 ± 441.6	164.8 ± 293.0	0.993
Total bilirubin, mg/dL	1.43 ± 1.87	1.44 ± 1.68	1.42 ± 2.17	0.915
**Albumin, g/dL**	2.8 ± 0.6	2.9 ± 0.6	2.6 ± 0.6	<0.001
BUN, mg/dL	39.9 ± 28.8	38.6 ± 30.0	42.1 ± 26.6	0.406
Creatinine, mg/dL	2.02 ± 1.73	2.00 ± 1.67	2.06 ± 1.84	0.783
C-reactive protein, ng/mL	15.7 ± 9.3	15.6 ± 8.9	16.1 ± 10.0	0.709
Procalcitonin, ng/mL	31.7 ± 47.5	31.5 ± 41.2	32.2 ± 57.1	0.915
Presepsin, pg/mL	1830.7 ± 2568.6	1813.9 ± 2974.1	1860.1 ± 1664.7	0.900
**Lactate, mmol/L**	3.83 ± 2.99	3.49 ± 2.58	4.42 ± 3.53	0.044

Data are presented as median and interquartile range, unless otherwise indicated.

BUN: blood urea nitrogen, ECOG: Eastern Cooperative Oncology Group, SAPS: Simplified Acute Physiology Score, SOFA: Sequential Organ Failure Assessment

### Patient prognosis and interventions

The patient prognoses and interventions are presented in Table 3. Non-survivors had higher rates of vasopressor (96.2% vs. 87.4%, p = 0.035) and inotrope (21.8% vs. 11.1%, p = 0.036) use than survivors. Non-survivors were more likely to receive invasive mechanical ventilation (80.8% vs. 35.6%, p < 0.001) and continuous renal replacement therapy (61.5% vs. 17.8%, p < 0.001) in the ICU. Although ECMO and hemodialysis were more prevalent in non-survivors, the differences were not statistically significant. Life-sustaining treatment decisions were observed more frequently in non-survivors than in survivors (93.6% vs. 36.3%, p < 0.001).

**Table 3:** Prognosis and interventions of the patients.

Characteristics	All patients	Survivor	Non-survivor	P-value
Patients (n)	213	135	78	
**Vasopressors**	193 (90.6)	118 (87.4)	75 (96.2)	0.035
**Inotropes**	32 (15.0)	15 (11.1)	17 (21.8)	0.036
Interventions in the ICU				
**Invasive mechanical ventilation**	111 (52.1)	48 (35.6)	63 (80.8)	<0.001
NIV	1 (0.5)	1 (0.7)	0 (0)	0.446
HFNC	94 (44.1)	63 (46.7)	31 (39.7)	0.327
**Continuous renal replacement therapy**	72 (33.8)	24 (17.8)	48 (61.5)	<0.001
ECMO	2 (0.9)	0 (0)	2 (2.6)	0.062
Hemodialysis	1 (0.5)	0 (0)	1 (1.3)	0.187
Tracheostomy	18 (8.5)	15 (11.1)	3 (3.8)	0.066
Clinical outcomes				
**ICU mortality**	63 (29.6)	0 (0)	63 (80.8)	<0.001
ICU stay, days	7.0 (3.0–15.0)	6.0 (3.0–12.0)	8.5 (2.0–21.0)	0.090
Hospital stay, days	16.0 (8.0–33.0)	19.0 (10.0–37.0)	10.0 (3.0–26.0)	0.268
**Life sustaining treatment issue**	122 (57.3)	49 (36.3)	73 (93.6)	<0.001
Appropriateness of antibiotics use within 24 hrs	33 (15.5)	18 (13.3)	15 (19.2)	0.252

Data are presented as median and interquartile range or number (%) unless otherwise indicated.

ICU: intensive Care Unit, NIV: Non-Invasive Ventilation, HFNC: high flow nasal cannula, ECMO: extracorporeal membrane oxygenation.

### Factors associated with mortality in the ICU and hospital

**Fig 2:**
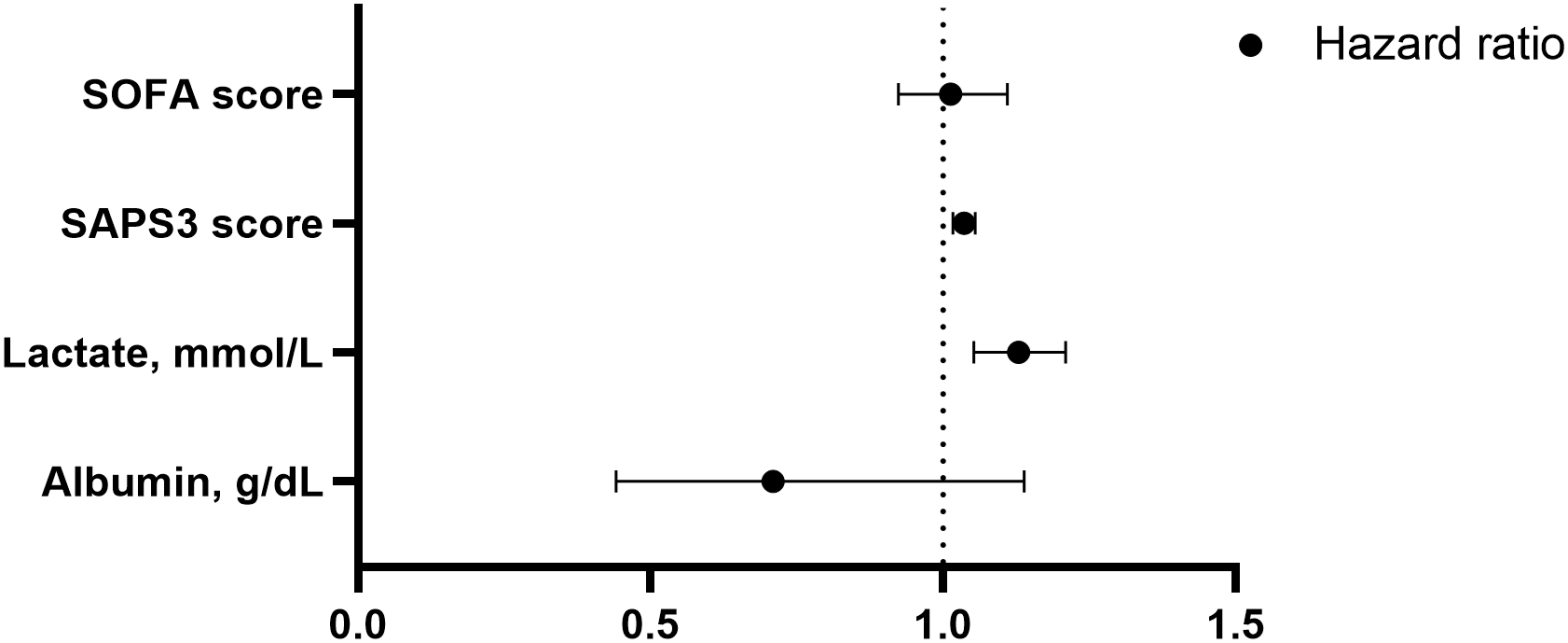
Forest plot of factors independently associated with ICU mortality in multivariate Cox regression analysis. The plot shows adjusted hazard ratios (HR) and 95% confidence intervals for key laboratory and clinical predictors. The vertical dashed black line represents the null (HR = 1). SAPS: Simplified Acute Physiology Score, SOFA: Sequential Organ Failure Assessment.

Table 4 and Fig 2 presents the factors associated with ICU mortality. Factors associated with ICU mortality were identified using patient laboratory results and scores. Univariate analysis identified low albumin levels, high lactate, high SAPS3 score, and high SOFA scores as factors associated with ICU mortality. Multivariate analysis further showed that high lactate levels (HR, 1.129; 95% CI, 1.053–1.210; p = 0.001) and high SAPS 3 scores (HR: 1.036, 95% CI, 1.017–1.055; p < 0.001) were independently associated with ICU mortality.

**Table 4:** Univariate and multivariate cox regression analysis addressing the factors for ICU mortality.

	Univariate analysis			Multivariate analysis		
	HR	95% CI	P-value	HR	95% CI	P-value
Laboratory findings						
White blood cell, × 10^3^/uL	0.985	0.955–1.016	0.339			
Neutrophil lymphocyte ratio	0.998	0.985–1.012	0.817			
Platelet, × 10^3^/uL	0.998	0.996–1.001	0.230			
Platelet lymphocyte ratio	1.000	0.999–1.000	0.476			
Albumin, g/dL	0.635	0.402–1.002	0.051	0.709	0.441–1.139	0.155
C-reactive protein, ng/mL	1.023	0.996–1.050	0.102			
Procalcitonin, ng/mL	1.003	0.998–1.008	0.188			
Presepsin, pg/mL	1.000	1.000–1.000	0.654			
**Lactate, mmol/L**	1.150	1.077–1.228	<0.001	1.129	1.053–1.210	0.001
Scores						
Charlson Comorbidity index	1.015	0.928–1.110	0.743			
Clinical frailty scale	1.099	0.929–1.298	0.271			
ECOG performance status	1.165	0.890–1.525	0.267			
**SAPS3 score**	1.040	1.022–1.059	<0.001	1.036	1.017–1.055	<0.001
SOFA score	1.110	1.021–1.206	0.014	1.013	0.924–1.110	0.790

HR: Hazard ratio, CI: Confidence interval, ECOG: Eastern Cooperative Oncology Group, SAPS: Simplified Acute Physiology Score, SOFA: Sequential Organ Failure Assessment.

Kaplan–Meier survival analyses were performed using optimal cutoff values determined by Youden’s index (S1 Fig). The median ICU follow-up duration was 7.0 days (IQR: 3.0–15.0). Patients with SAPS 3 ≥ 70.5 had a median survival of 23.0 days versus 58.0 days for those with SAPS 3 < 70.5 (log-rank p = 0.0011; HR = 2.743, 95% CI: 1.608–4.681). Similarly, patients with lactate ≥4.55 mmol/L had a median survival of 23.0 days versus 49.0 days (log-rank p = 0.0017; HR = 2.205, 95% CI: 1.253–3.880). The proportional hazards assumption for the ICU mortality Cox model was tested using Schoenfeld residuals. No significant violation was found for the covariates (SAPS 3, p = 0.084; lactate, p = 0.028), although the global test indicated a marginal violation (χ² = 6.81, df = 2, p = 0.033). The Schoenfeld plots showed no clear systematic deviation from proportionality (S2 Fig).

**Fig 3:**
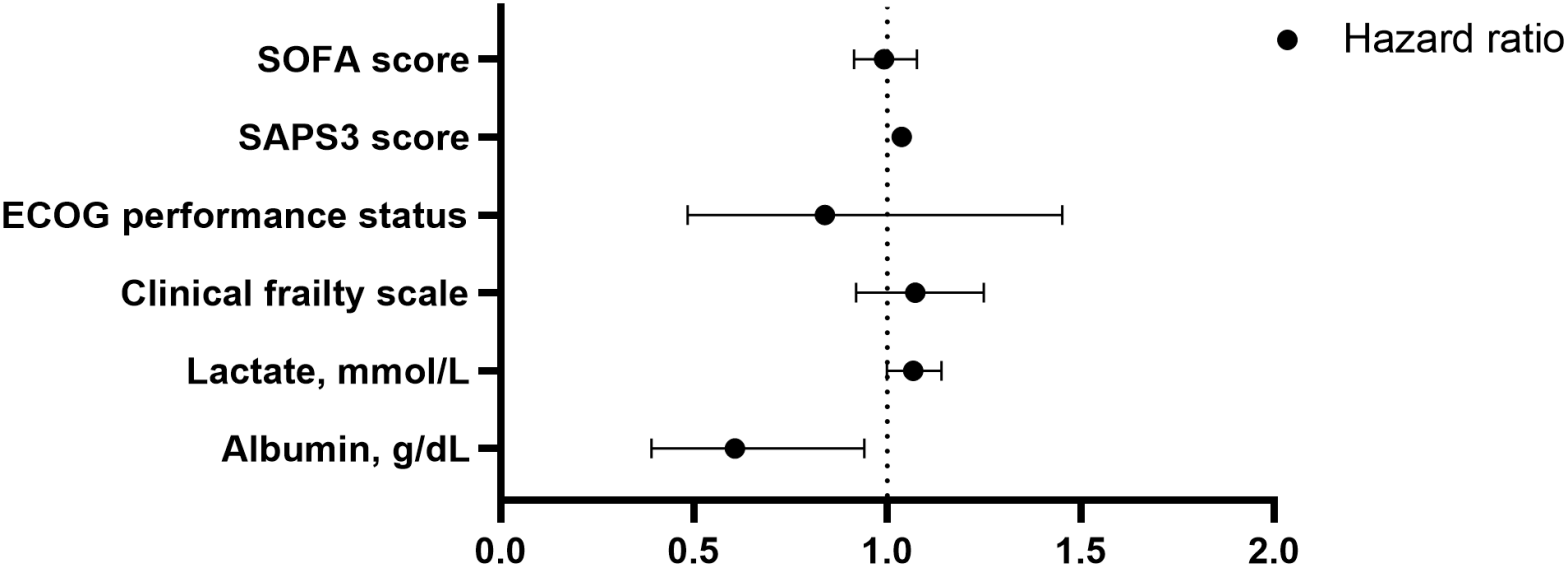
Forest plot of factors independently associated with in-hospital mortality in multivariate Cox regression analysis. The plot shows adjusted hazard ratios (HR) and 95% confidence intervals for key laboratory and clinical predictors. The vertical dashed black line represents the null (HR = 1). ECOG: Eastern Cooperative Oncology Group, SAPS: Simplified Acute Physiology Score, SOFA: Sequential Organ Failure Assessment.

The factors associated with in-hospital mortality are shown in Table 5 and Fig 3. Patient laboratory results and scores were used to identify factors associated with in-hospital mortality. Univariate analysis identified low albumin, high lactate, high CFS, high ECOG, high SAPS 3, and high SOFA scores as factors associated with in-hospital mortality. Multivariate analysis further showed that low albumin levels (HR: 0.606, 95% CI: 0.390–0.940, p = 0.025) and high SAPS 3 score (HR: 1.037, 95% CI: 1.021–1.054, p < 0.001) were independently associated with in-hospital mortality. High lactate levels also showed a potential association (HR, 1.067; 95% CI, 0.999–1.140; p = 0.055).

**Table 5:** Univariate and multivariate cox regression analysis addressing the factors for in-hospital mortality.

	Univariate analysis			Multivariate analysis		
	HR	95% CI	P-value	HR	95% CI	P-value
Laboratory findings						
White blood cell, × 10^3^/uL	0.986	0.961–1.012	0.285			
Neutrophil lymphocyte ratio	0.995	0.984–1.007	0.426			
Platelet, × 10^3^/uL	0.999	0.997–1.002	0.573			
Platelet lymphocyte ratio	1.000	0.999–1.001	0.773			
**Albumin, g/dL**	0.554	0.367–0.837	0.005	0.606	0.390–0.940	0.025
C-reactive protein, ng/mL	1.006	0.982–1.031	0.620			
Procalcitonin, ng/mL	0.999	0.994–1.005	0.846			
Presepsin, pg/mL	1.000	1.000–1.000	0.783			
Lactate, mmol/L	1.096	1.026–1.172	0.006	1.067	0.999–1.140	0.055
Scores						
Charlson Comorbidity index	1.036	0.958–1.121	0.379			
Clinical frailty scale	1.183	1.016–1.377	0.030	1.072	0.919–1.250	0.376
ECOG performance status	1.239	0.983–1.562	0.069	0.839	0.484–1.453	0.530
**SAPS3 score**	1.041	1.025–1.058	<0.001	1.037	1.021–1.054	<0.001
SOFA score	1.090	1.017–1.168	0.015	0.992	0.914–1.077	0.851

HR: Hazard ratio, CI: Confidence interval, ECOG: Eastern Cooperative Oncology Group, SAPS: Simplified Acute Physiology Score, SOFA: Sequential Organ Failure Assessment

Kaplan-Meier survival curves stratified by SAPS 3 and albumin levels are shown in S3 Fig. The median follow-up duration was 16.0 days (IQR: 8.0–33.0). Patients with SAPS 3 ≥ 70.5 had a median survival of 30.0 days versus not reached in the low-risk group (p < 0.0001; HR: 3.037, 95% CI: 1.913–4.822). Patients with albumin <2.65 g/dL had a median survival of 41.0 days versus 177.0 days in the higher albumin group (p = 0.0035; HR: 0.507, 95% CI: 0.320–0.813). For the in-hospital mortality Cox model, the proportional hazards assumption was not violated according to the Schoenfeld test (SAPS 3, p = 0.149; albumin, p = 0.108; global, p = 0.095). The corresponding residual plots are shown in S4 Fig.

## Discussion

This study evaluated the prognostic value of inflammatory markers and clinical scoring systems in predicting ICU and hospital mortality in patients with sepsis. Non-survivors had higher levels of inflammatory markers such as lactate and lower levels of albumin compared to survivors. While CRP, presepsin, and procalcitonin levels tended to be higher in non-survivors, they were not independently associated with mortality in multivariate analyses, suggesting their limited predictive value when adjusted for other factors.

Among the scoring systems evaluated, SAPS 3, SOFA, CCI, CFS, and ECOG performance status were all significantly increased in non-survivors. On multivariate analysis, SAPS 3 emerged as the most consistent and independent predictor of both ICU and in-hospital mortality. This finding is consistent with previous studies demonstrating the strong prognostic performance of SAPS 3 in sepsis populations. Zhu et al. [11] demonstrated that SAPS 3 was effective in stratifying mortality risk in critically ill patients with sepsis, while Stoiber et al. [18] showed that combining SAPS 3 with lactate levels improved prediction of 28-day mortality in patients with septic shock.

In our cohort, elevated lactate levels were independently associated with ICU mortality. Lactate is a well-established marker of tissue hypoperfusion and cellular dysfunction, and our findings support its continued use in early risk assessment. Previous studies have reported a strong association between hyperlactatemia and poor outcomes. For example, Chertoff et al. [19] discussed lactate as an indicator of metabolic stress in septic patients, while Filho et al. [20] identified an initial lactate ≥2.5 mmol/L as a strong predictor of 28-day mortality in severe sepsis. Mikkelsen et al. [21] further demonstrated that both intermediate (2–3.9 mmol/L) and high (≥4 mmol/L) lactate levels were associated with increased mortality, regardless of shock or organ failure status.

Low albumin levels were independently associated with in-hospital mortality in our analysis. Albumin reflects both nutritional status and the degree of systemic inflammation. Our findings are supported by Takegawa et al. [22], who showed that reductions in nutritional markers such as albumin were strongly associated with mortality in septic patients. Similarly, Eskart et al. [23] found that hypoalbuminemia was associated with systemic inflammation and poor outcome in critical illness. Turcato et al. [24] and Cao et al. [25] also identified admission serum albumin as a reliable predictor of 30-day and long-term mortality in patients with sepsis, with thresholds such as <2.6 g/dL associated with poor prognosis.

Frailty and comorbidities also significantly influenced patient outcomes. In our study, higher CCI and CFS scores were observed in non-survivors, highlighting the role of preexisting health status in sepsis prognosis. Komori et al. [26] reported that frailty was significantly associated with increased mortality in patients with suspected infection, which is consistent with our findings regarding CFS. In addition, a large cohort study [27] confirmed that higher CCI scores were associated with increased sepsis-related mortality.

Of the evaluated scoring systems, SAPS 3 was the most consistent independent predictor of ICU and in-hospital mortality in the multivariate analysis. An optimal cutoff of 70.5 demonstrated good sensitivity (82.5% for ICU mortality and 78.2% for in-hospital mortality) and reasonable specificity (62.0% and 64.4%, respectively), supporting its practical utility for risk stratification. These results are consistent with those of Zhu et al. [11], who found that SAPS 3 had the greatest discriminative ability for predicting 28-day mortality in patients with sepsis (AUROC = 0.812), outperforming SOFA and other models significantly. Additionally, Czajka et al. [28] demonstrated that physiology-based scoring systems maintain acceptable predictive accuracy across diverse ICU settings. SAPS 3’s robust performance across ICU and hospital outcomes underscores its clinical value as a prognostic tool for critically ill sepsis patients, supporting its routine implementation in early ICU assessments.

Our study highlights the importance of integrating acute phase markers (e.g., lactate), baseline status indicators (e.g., albumin), and validated clinical scoring systems (e.g., SAPS 3) to improve risk stratification and inform clinical decision making in sepsis. These tools support early prognostic assessment, guide treatment decisions, and help identify patients who may benefit from targeted interventions. For example, hypoalbuminemia could serve as a potential target for nutritional support or albumin supplementation strategies, warranting further investigation in interventional trials. In addition, the utility of SAPS 3 should be validated in different ICU populations, including resource-limited settings and specific subgroups of patients with sepsis. From a practical standpoint, ICU protocols could be adapted to incorporate early SAPS 3 scoring at the time of admission and routine lactate monitoring during the initial sepsis assessment. Incorporating these assessments into standardized workflows may facilitate timely risk stratification, allow for earlier clinical intervention, and optimize resource allocation in critically ill patients.

Although presepsin was evaluated in our study, it was not independently associated with mortality. However, it remains a promising biomarker for early diagnosis and risk stratification of sepsis, as suggested by previous research [29]. Future studies should further investigate its clinical utility in combination with established tools. In addition, other emerging biomarkers-such as proadrenomedullin [30], soluble urokinase plasminogen activator receptor [31], and interleukin-6 [32] may provide insight into the severity of infection, immune dysregulation, or endothelial dysfunction and warrant further investigation. Incorporating these markers into prognostic models could improve the precision and personalization of sepsis management and inform future research directions.

This study has several limitations. First, due to its retrospective design, selection and information bias may have influenced the results. Specifically, there is a possibility of residual confounding because not all relevant clinical variables, such as timing of antibiotics or vasopressor initiation, could be fully captured or adjusted for the analysis. Second, data were collected from a single university-affiliated hospital, which may limit the generalizability of the findings. Our study population consisted predominantly of older adults treated in a Korean tertiary care setting, and the demographic and clinical characteristics may differ from those in other regions or health care systems. Third, although we adjusted several confounding variables, unmeasured factors-such as ICU staffing levels, treatment protocols, or clinical decision making-may have influenced the results. Finally, reliance on electronic medical records may have led to incomplete data collection, particularly for variables such as medication use and timing of clinical interventions, which could have introduced misclassification or affected the strength of the observed associations.

## Conclusion

This study found that elevated lactate levels and high SAPS 3 scores were independently associated with ICU mortality. Meanwhile, low albumin levels and high SAPS 3 scores were associated with in-hospital mortality in patients with sepsis. Traditional inflammatory markers, such as CRP and procalcitonin, did not demonstrate independent prognostic value. These results suggest that combining physiological markers with clinical scoring systems could be useful for assessing risk in sepsis patients. Further validation studies are needed to confirm these findings and evaluate their clinical utility.

## Supporting information

S1 Fig Kaplan–Meier survival curves stratified by (A) SAPS 3 (≥70.5 vs. < 70.5; sensitivity 82.5%, specificity 62.0%) and (B) lactate (≥4.55 vs. < 4.55 mmol/L; sensitivity 54.0%, specificity 68.0%). Both higher SAPS 3 and lactate levels were associated with significantly reduced ICU survival (p < 0.01). Cutoff values were determined using Youden’s index.
(TIF)

S2 Fig Schoenfeld residual plots for SAPS 3 and lactate in the Cox model predicting ICU mortality. (TIF)

S3 Fig Kaplan–Meier curves for in-hospital mortality stratified by (A) SAPS 3 (≥70.5 vs. < 70.5; sensitivity: 78.2%, specificity: 64.4%) and (B) albumin (<2.65 vs. ≥ 2.65 g/dL; sensitivity: 38.5%, specificity: 66.7%). Higher SAPS 3 and lower albumin were associated with significantly higher mortality (p < 0.001 and p = 0.004, respectively). Cutoff values were determined using Youden’s index.
(TIF)

S4 Fig Schoenfeld residual plots for SAPS 3 and albumin in the Cox model predicting in-hospital mortality. (TIF)
