# Supplementary figures and images for: Prognostic value of laboratory markers and clinical scores for mortality in intensive care unit patients with sepsis
Source: PLoS One. 2025 Dec 4;20(12):e0337396. doi: 10.1371/journal.pone.0337396 (PMC12677509; doi:10.1371/journal.pone.0337396)

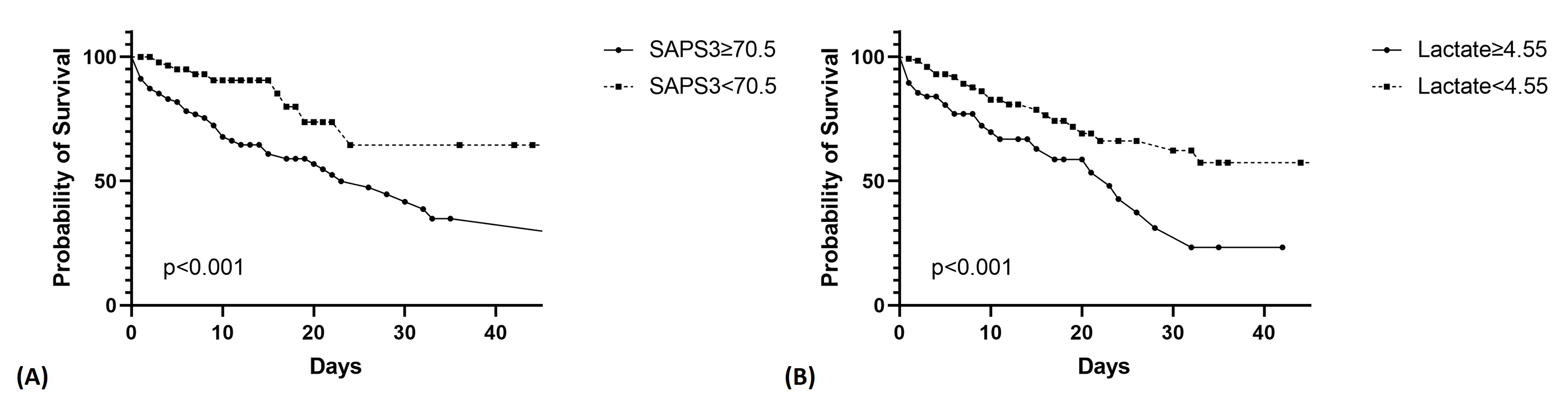

Supplement: S1 Fig — Both higher SAPS 3 and lactate levels were associated with significantly reduced ICU survival (p < 0.01). Cutoff values were determined using Youden’s index. (TIF) [file pone.0337396.s001.tif]

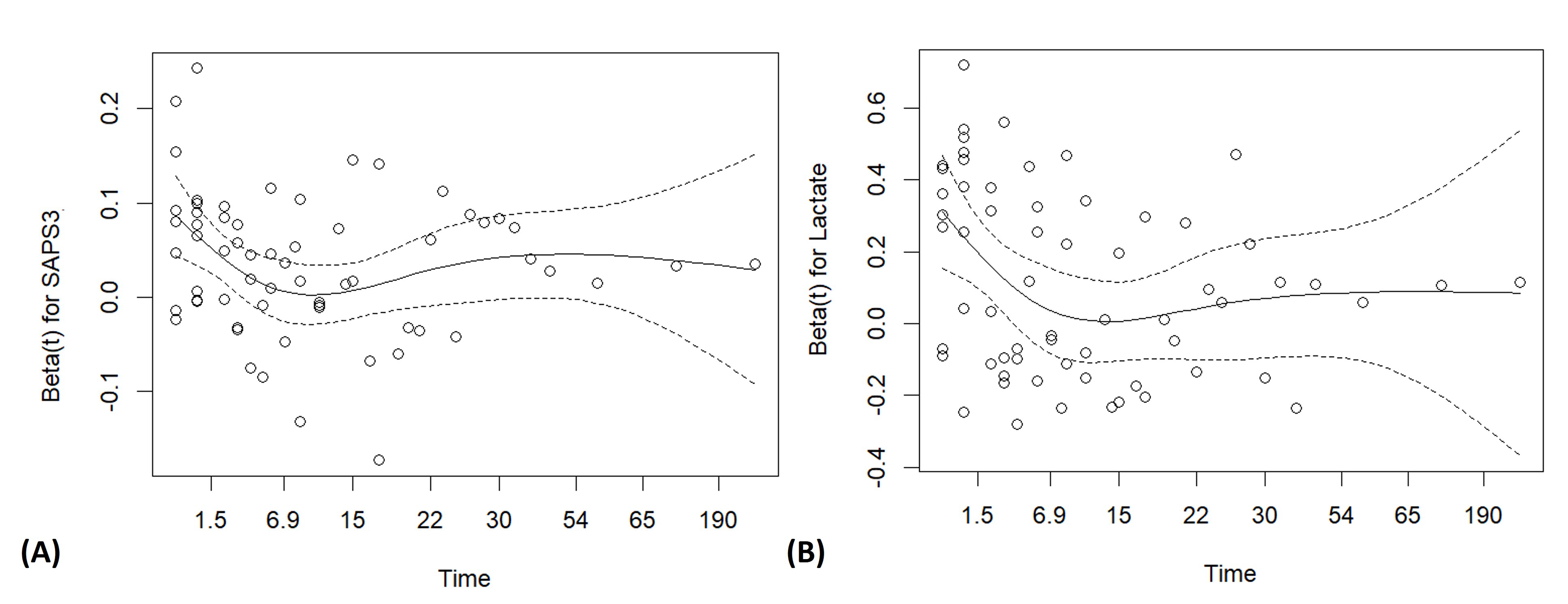

Supplement: S2 Fig — (TIF) [file pone.0337396.s002.tif]

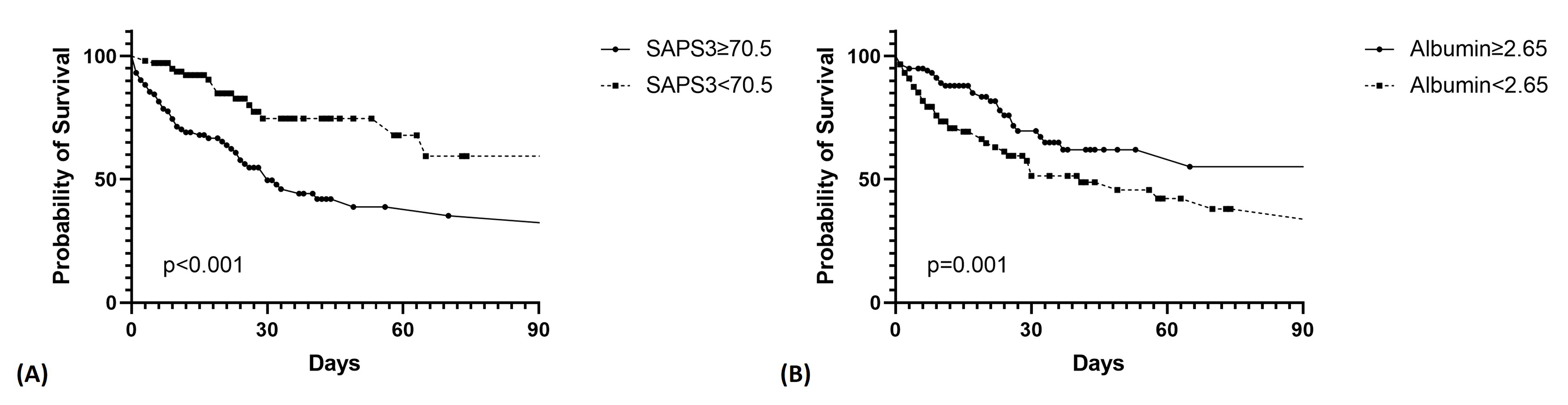

Supplement: S3 Fig — Higher SAPS 3 and lower albumin were associated with significantly higher mortality (p < 0.001 and p = 0.004, respectively). Cutoff values were determined using Youden’s index. (TIF) [file pone.0337396.s003.tif]

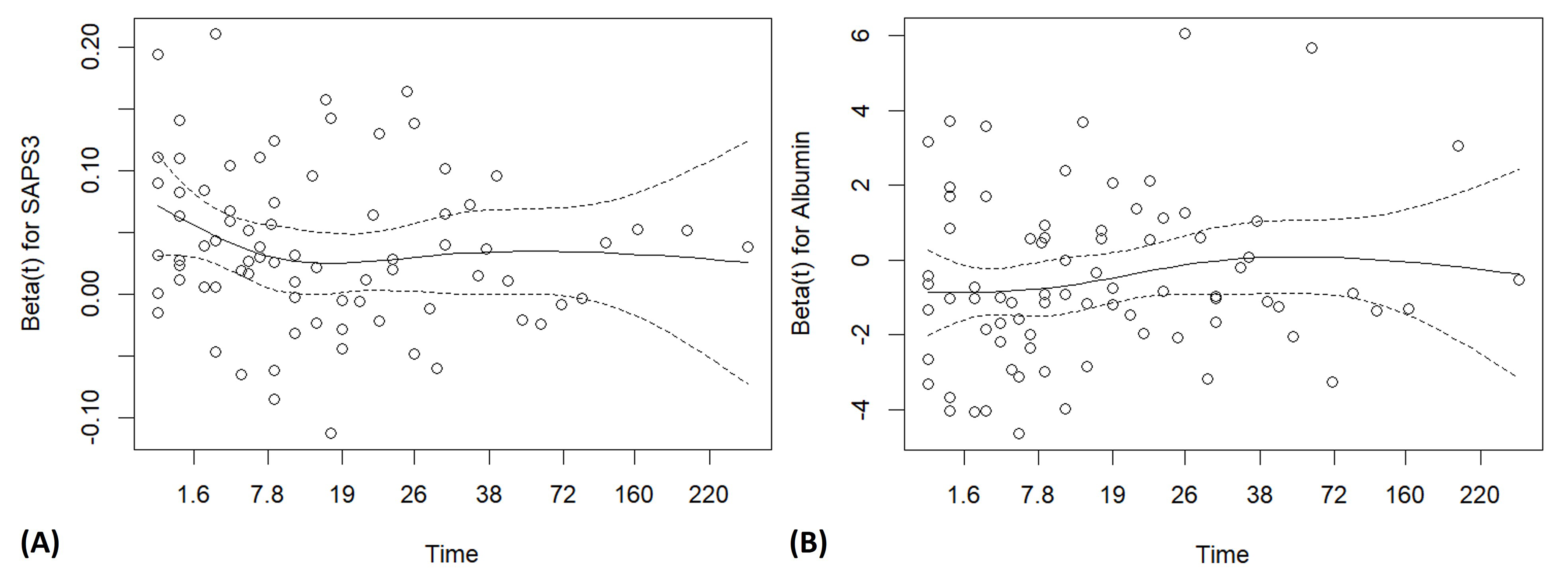

Supplement: S4 Fig — (TIF) [file pone.0337396.s004.tif]
